# Cdc20 directs proteasome-mediated degradation of the tumor suppressor SMAR1 in higher grades of cancer through the anaphase promoting complex

**DOI:** 10.1038/cddis.2017.270

**Published:** 2017-06-15

**Authors:** Debasish Paul, Suvankar Ghorai, U S Dinesh, Praveenkumar Shetty, Samit Chattopadhyay, Manas Kumar Santra

**Affiliations:** 1 National Centre for Cell Science, Pune, Maharashtra, India; 2 Department of Pathology, SDM College of Medical Sciences & Hospital, Dharwad, Karnataka, India; 3 Department of Biochemistry/Central research laboratory, SDM College of Medical Sciences & Hospital, Dharwad, Karnataka, India; 4 Indian Institute of Chemical Biology, Kolkata, West Bengal, India

## Abstract

The Tumor suppressor SMAR1 (scaffold matrix attachment region binding protein 1) has a crucial role in maintaining genomic stability, cell cycle progression and apoptosis.Our previous finding showed that it is highly suppressed in higher grade of cancer. However, the underlying mechanism of this suppression was not well understood. In this study, we show that SMAR1 expression levels are controlled at the proteasomal level by five RING finger E3 ubiquitin ligases including, Cdc20, a substrate receptor of ubiquitin ligase APC/C complex. We found that Cdc20 binds and promotes proteasomal degradation of SMAR1 in a D-box motif dependent manner. Further, our results demonstrated that Cdc20 promotes proteasomal degradation of SMAR1 through K48-linked specific polyubiquitylation, and that short hairpin RNA mediated inactivation of Cdc20 leads to significant stabilization of SMAR1. These findings suggest that Cdc20 is responsible for maintaining the cellular levels of SMAR1. However, since Cdc20 fails to target SMAR1 upon exposure to genotoxic stresses, SMAR1 helps to maintain genomic stability under these conditions through its DNA damage repair activity. Interestingly, Cdc20-mediated degradation of SMAR1 promotes cell migration and invasion.The reciprocal relationship of the duo is evident in breast cancer cell lines as well as in patient samples, suggesting that Cdc20 functions as an important negative regulator of SMAR1 in higher grades of cancer. Our study reveals for the first time, the molecular mechanism associated with lower levels of expression of the important tumor suppressor SMAR1 in higher grades of breast cancer.

Scaffold/matrix attachment regions (S/MARs), belong to the class of regulatory DNA elements, are mostly present upstream of promoter sequences. SMAR1 (scaffold matrix attachment region binding protein 1) is a MAR-binding protein first identified in mouse, which shows >95% homology with its human counterpart BANP.^1, 2^ It was earlier reported that SMAR1 acts as a potential tumor suppressor by arresting cells at the G1 and G2/M phases of the cell cycle through activation of p53.^3^ SMAR1 is also reported to be involved in suppression of metastasis and DNA damage repair pathway.^4, 5, 6^ Recent report have shown that SMAR1 functions as a tumor suppressor by preventing the formation of the oncogenic form of CD44 by altering the splicing.^7^ SMAR1 is reported to be highly suppressed in higher grades of cancer.^8^ Though SMAR1 is known to be partially inactivated through the loss of heterozygosity (LOH),^9^ the exact mechanism of its regulation in normal and cancer cells is largely unknown.

Many tumor suppressors are inactivated through multiple mechanisms such as epigenetic gene silencing, LOH, mutation and proteasomal deregulation. For example, the cellular levels of the well-known tumor suppressor p53,are maintained at the proteasomal level through RING finger E3 ubiquitin ligases.^10^ Interestingly, the majority of cellular proteins are regulated at the proteasomal level mostly through the Ring-finger E3 ubiquitin ligase, SCF and/or anaphase-promoting complex/cyclosome (APC/C) complex.

APC/C is a multi protein complex has an important role in the progression of the G2/M and G1 phases of the cell cycle through selective proteasomal degradation of cell cycle regulatory proteins.^11^ The substrate receptor subunit Cdc20 (cell division cycle 20 homolog) and Cdh1 of the APC/C complex mostly recognize the D-box (RXXL) and/or KEN motif.^12^ APC/C^Cdc20^ has important roles in cell cycle progression through proteasomal degradation of many proteins, including Nek2A and cyclin A, at the transition from prophase to prometaphase, and promotes degradation of cyclin B and securin during the metaphase to anaphase transition.^13, 14, 15^ Cdc20 expression has been reported to be significantly elevated in higher grades of cancers and has been linked to poor prognosis in pancreatic, lung, bladder, colon, oral squamous cell carcinomas and breast cancer.^16, 17, 18, 19, 20, 21^

In this study, we have investigated the proteasomal regulation of SMAR1 in breast cancer. We have shown that cellular levels of SMAR1 are regulated at the proteasomal level through APC/C^Cdc20^.Cdc20 interacts by recognizing the D-box motif and promotes lysine48-linked polyubiquitylation-mediated proteasomal degradation of SMAR1 in an APC/C dependent manner, a process prevented by the cellular kinase JNK. However, Cdc20 fails to target SMAR1 for proteasomal degradation upon exposure genotoxic stress, suggesting that Cdc20 limits the cellular function of SMAR1 only in normal cells. Further, our study revealed that Cdc20 accelerates cell migration and invasion through limiting the expression of SMAR1. Interestingly, a converse relationship of Cdc20 and SMAR1 was observed in breast cancer patient samples, with under expression of SMAR1 in higher grades supporting that oncogenic Cdc20 limits SMAR1 levels in higher grade of breast cancer. Collectively our findings reveal, for the first time, the intriguing molecular mechanism of inactivation of the SMAR1 in higher grades of cancer, suggesting that the use of small molecules that can prevent SMAR1-Cdc20 interactions might be a good strategy for chemotherapy.

## Results

### SMAR1 is regulated via proteasomal pathway

**Figure 1:**
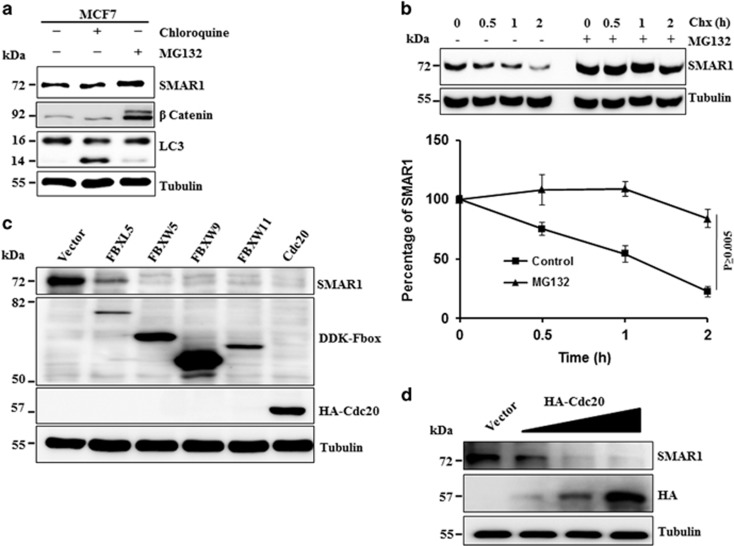
SMAR1 is regulated via proteasomal pathway. (**a**) Endogenous level of SMAR1 is stabilized upon MG132 treatment. MCF7 cells were treated with 10 *μ*M MG132 (proteasome inhibitor) and 100 *μ*M Chloroquine (lysosome inhibitor) for 6 h. Cells were collected, prepared whole protein extracts and immunoblotted for indicated proteins. (**b**) MG132 alters the turnover kinetics of SMAR1. MCF7 cells were treated with 40 *μ*g/ml cycloheximide in the absence and presence of 10 *μ*M MG132 and treated cells were harvested at indicated time points. Whole cell protein extracts were analyzed by immunoblotting with indicated antibodies. Expression level of each protein on immunoblot (**b**) was quantified densitometrically. Then, expression of SMAR1 at each time point was normalized with tubulin expression and presented graphically. (**c**) Suppression of SMAR1 expression by candidate genes. Candidate genes were ectopically expressed in MCF7 cells for 48 h. Cells were then collected, prepared whole cell protein extracts and analyzed by immunoblotting with indicated antibodies. (**d**) Cdc20 degrades SMAR1 in a dose dependent manner. MCF7 cells were transfected with increasing doses of HA-Cdc20, prepared the protein extracts and immunoblotted for indicated proteins

Previous study has shown that SMAR1 levels are highly repressed in higher grades of cancer.^4^ Levels of tumor suppressors are kept down by different ways in cancer, such as LOH, epigenetic silencing and proteasomal deregulation. To understand whether SMAR1 is regulated at the posttranslational levels, MCF7 cells were treated with either MG132 (proteasome inhibitor) or chloroquine (lysosome inhibitor). SMAR1 was found to be significantly stabilized only upon inhibition of the proteasome system (Figure 1a). Further, SMAR1 was also significantly stabilized in multiple breast cancer, colorectal cancer HCT116, and cervical cancer HeLa cell line in the presence of MG132 (Supplementary Figures 1a–d). Moreover, the stabilization of SMAR1 was more prominent in highly metastatic breast cancer cell lines MDA-MB-231 (Triple negative breast cancer, Basal B), NCI-ADR-RES (Luminal A-metastatic) and T47D (Luminal A), than in MCF7 (Lower metastatic, luminal A) (Supplementary Figures 1a and b) following treatment with MG132.^22, 23, 24^ The cycloheximide pulse chase assay provided additional evidence that SMAR1 is regulated at the proteasomal level (Figure 1b). For example, SMAR1 half-life is increased more than two folds in the presence of MG132 (Figure 1b). Noticeably, the abundance of *SMAR1* mRNA was found to be unaltered following MG132 treatment (Supplementary Figure 1c). Collectively, results indicate that SMAR1 is regulated through proteasome.

Since RING-finger E3 ubiquitin ligases are mostly involved in proteasomal regulation of the cellular proteins, we performed SCF and APC/C E3 ubiquitin ligase screens and identified FBXL5, FBXW5, FBXW11, FBXW9 and Cdc20 as regulator of SMAR1 (Figure 1c). We selected Cdc20 for detailed studies because of its recognition D-box motifs (RXXL) present in SMAR1. We checked whether Cdc20 is a potent regulator of SMAR1 through increased ectopic expression of HA-Cdc20 and found that SMAR1 is significantly degraded with increasing expression of Cdc20 (Figure 1d), indicating that SMAR1 is a cellular target of Cdc20.

### Cdc20 regulates SMAR1 at the proteasomal level

**Figure 2:**
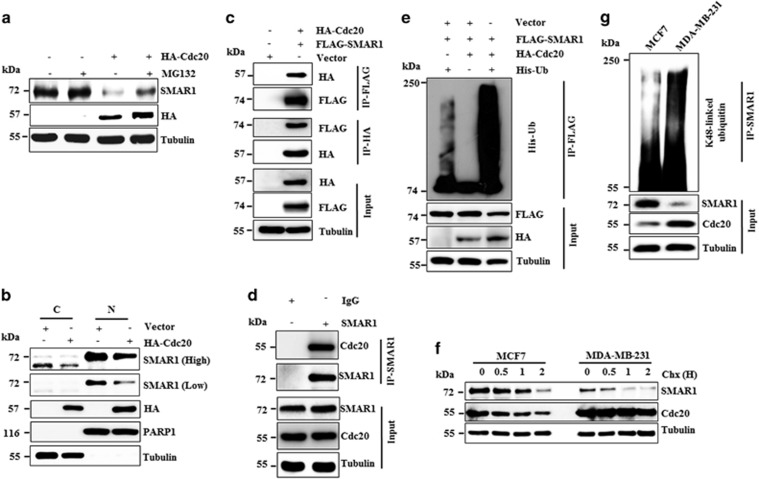
Cdc20 interacts with and promotes proteasomal degradation of SMAR1. (**a**) Cdc20 degrades SMAR1 through proteasome. MCF7 cells were transfected with either vector or HA-Cdc20 for 36 h and then treated with or without 10 *μ*M MG132 for 6 h. Cells were then harvested, prepared protein extracts and immunoblotted for indicated antibodies. (**b**) Cdc20 targets both cytoplasmic and nuclear fraction of SMAR1. MCF7 cells were transfected with either vector or HA-Cdc20 for 48 h. Cells were then collected and fractionated for cytoplasmic and nuclear pool followed by immunoblotting for indicated antibodies. (**c**) Cdc20 interacts with SMAR1. MCF7 cells were transfected either vector or combination of HA-Cdc20 and FLAG-SMAR1 for 36 h. Transfected cells were then treated with 10 *μ*M MG132 for 6 h. Whole cell protein extracts were used for immunoprecipitation (IP) with indicated antibody. The immune precipitates were then immunoblotted for indicated proteins. (**d**) SMAR1-Cdc20 interacts at the endogenous level. MCF7 cell extracts were immunoprecipitated with either IgG or SMAR1 antibody followed by immunoblotted for indicated proteins. (**e**) Cdc20 promotes polyubiquitylation of SMAR1 *in vivo*. MCF7 cells were co-transfected with indicated plasmids for 36 h.Transfected cells were then treated with 10 *μ*M MG132 for last 6 h. Whole cell protein extracts were immunoprecipitated with anti-FLAG antibody and immunoprecipitates were immunoblottedfor ubiquitin. (**f**) Cycloheximide pulse chase assay showed the differential turnover kinetics of SMAR1 in MCF7 and MDA-MB-231 cell lines. (**g**) Comparative polyubiquitination profile of SMAR1 in MCF7 and MDA-MB-231. MCF7 and MDA-MB-231 cells were treated with 10 *μ*M MG132 for 6 h and lysates were immunoprecipitated for SMAR1 and the immunoprecipitates were probed for anti-K48-linked ubiquitin

Next, we tested whether Cdc20 directs the degradation of SMAR1 through the proteasomal pathway. Immunoblot analysis showed that Cdc20 significantly degrades SMAR1, which was inhibited in the presence of MG132 (Figure 2a), suggesting that Cdc20 directs proteasomal degradation of SMAR1. Further studies revealed that Cdc20 degrades both the nuclear and cytoplasmic pool of SMAR1 (Figure 2b). Collectively, these results revealed that Cdc20 degrades SMAR1 through 26 S proteasome.

### Cdc20 directly interacts with SMAR1

Cdc20 regulates its substrates through direct interaction and we therefore investigated whether Cdc20 and SMAR1 interact through co-immunoprecipitation assay. Results demonstrated the presence of HA-Cdc20 in the immunoprecipitate of FLAG-SMAR1 (Figure 2c). Similar results were also observed in the reciprocal co-immunoprecipitation assay, confirming their interaction (Figure 2c). Similarly, Cdc20 was found to interact with SMAR1 at the endogenous level (Figure 2d).

### Cdc20 promotes the polyubiquitylation of SMAR1

The direct interaction of SMAR1-Cdc20 prompted us to examine whether Cdc20 polyubiquitylates SMAR1. MCF7 cells were co-transfected with FLAG-SMAR1, HA-Cdc20 and His-ubiquitin plasmids. Immunoblotting analysis of anti-FLAG immunoprecipitates showed the presence of ubiquitylated high mass ladder of SMAR1 only in the presence of Cdc20 and ubiquitin, suggesting that Cdc20 promotes polyubiquitylation of SMAR1 (Figure 2e). Further, Cdc20-mediated proteasomal turnover kinetics of SMAR1 was examined in MCF7 cells (lower Cdc20 levels) and MDA-MB-231 cells (higher Cdc20 levels) through cycloheximide pulse chase experiment. Results demonstrated that the turnover of SMAR1 is 2 fold higher in MDA-MB-231, as compared with MCF7 cells (Figure 2f and Supplementary Figure 2). For instance, the half-life of SMAR1 is 80±07 and 40±05 min in MCF7 and MDA-MB-231 cells, respectively. This difference in the half-life of SMAR1 is in good agreement with cellular levels of Cdc20 in these cell lines (Figure 2f). In agreement with the turnover kinetics, we observed higher levels of K48 (lysine 48 of ubiquitin)-linked polyubiquitylated SMAR1 in MDA-MB-231 as compared with MCF7 cells (Figure 2g). These results collectively suggest that the SMAR1 protein levels are significantly reduced in higher grades of breast cancer due to proteasomal degradation by Cdc20.

### Cdc20-mediated SMAR1 degradation is D-box dependent

**Figure 3:**
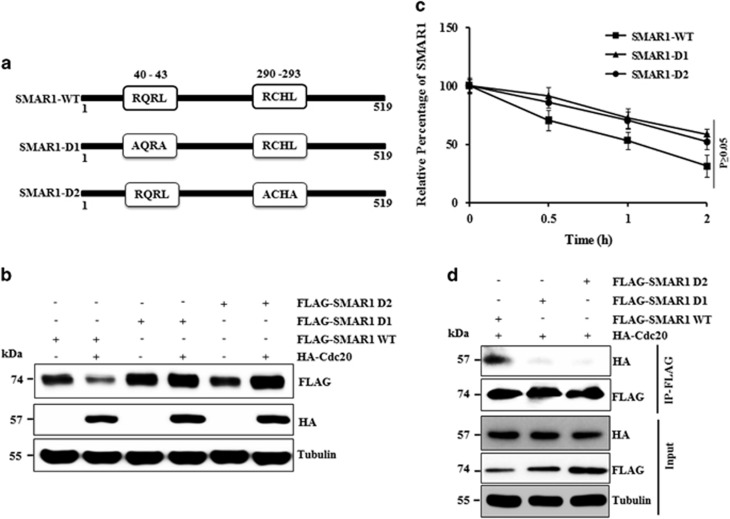
Degradation of SMAR1 by Cdc20 is D-box dependent. (**a**) Schematic representation of D-box location in SMAR1. First D-box is located in 40–43aa and second D-box in 290–293aa location. Schematic diagram showing mutation created in both the D-boxes. (**b**) Cdc20-mediated SMAR1 degradation is D box dependent. FLAG-SMAR1-WT or FLAG-SMAR1-D1 or FLAG-SMAR1-D2 were co-transfected either with vector or HA-Cdc20 in MCF7 cells. Whole-cell lysates were immunoblotted with indicated antibodies. (**c**) MCF7 cells were transfected with FLAG-SMAR1-WT and FLAG-SMAR1-D1 and FLAG-SMAR1-D2 mutant. Transfected cells were pulsed with cycloheximide (40 *μ*g/ml) and chased for indicated time points. Expression level of SMAR1-WT, SMAR1-D1 and SMAR1-D2 and tubulin on immunoblot were quantified densitometrically and presented graphically after normalization at zero time point. (**d**) Cdc20 fails to interact with SMAR1 D-box mutants. MCF7 cells were co-transfected with FLAG-SMAR1-WT, FLAG-SMAR1-D1, FLAG-SMAR1-D2 and HA-tagged Cdc20. Cell lysate was immunoprecipitated with anti-FLAG antibody and immunoblotted for indicated proteins

Previous studies have identified D-box (RXXLXXXXN/RXXL) as the substrate-recognition motif of Cdc20.^11^ We found the presence of two highly conserved D-box motifs in SMAR1 (40–43aa and 290–293aa) (Figure 3a; Supplementary Figure 3). To understand whether the D-box motif of SMAR1 is required for Cdc20-mediated degradation, two D-box mutants of SMAR1 (D1 and D2) were generated (Figure 3a). MCF7 cells were transfected with wild-type SMAR1 (SMAR1-WT), SMAR1-D1 and SMAR1-D2 either with vector or HA-Cdc20. Immunoblotting data revealed that Cdc20 significantly degrades SMAR1-WT but fails to degrade both SMAR1-D1 and SMAR1-D2 (Figure 3b). Moreover, cycloheximide pulse chase experiments showed an increase in half-life (1.5 times) of D-box mutants, as compared with SMAR1-WT (Figure 3c). To understand why Cdc20 failed to degrade mutant SMAR1, we examined their interaction through coimmunoprecipitation assay. The results demonstrated that Cdc20 is immunoprecipitated only with SMAR1-WT, suggesting that both the D-box motifs of SMAR1 are essential for its interaction with Cdc20 (Figure 3d).

### Cellular levels of SMAR1 are regulated by Cdc20

**Figure 4:**
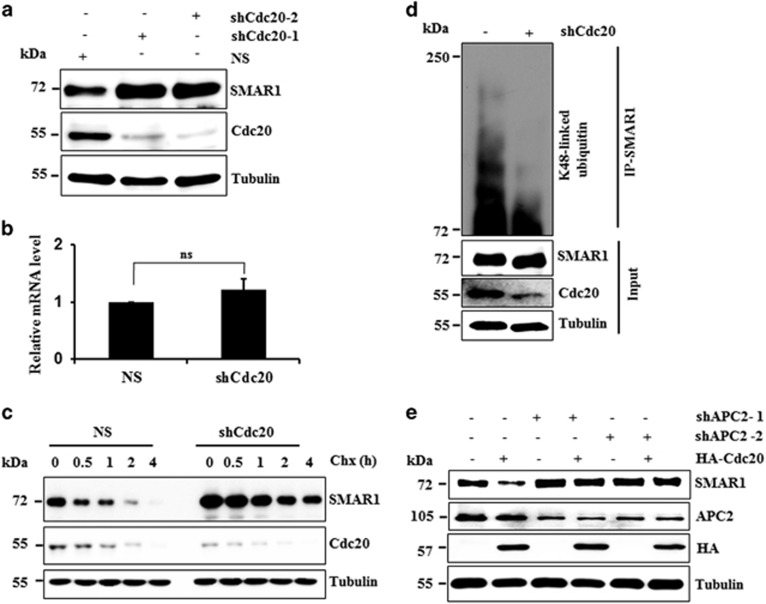
Cdc20 regulates cellular levels of SMAR1 through APC/C complex. (**a**) SMAR1 is significantly stabilized in Cdc20KD cells. Stable knockdown cells of Cdc20 was made by specific shRNAs against Cdc20 (shCdc20) in MCF7 and scramble shRNA (NS) was used as control and lysates were immunoblotted for indicated proteins. (**b**) Relative mRNA level of SMAR1 in Cdc20KD and NS cells. SMAR1 mRNA levels were measured using RT-qPCR assay and normalized against GAPDH mRNA. NS represents statistically not significant difference calculated by one way ANOVA test (**c**) Slower turnover of SMAR1 in Cdc20KD cells. NS or Cdc20KD cells were treated with cycloheximide (40 *μ*g/ml) for indicated periods and then cells were harvested, prepared cell lysates and immunoblotted for indicated proteins. (**d**) Cdc20 promotes lysine 48-linked polyubiquitylation of SMAR1. NS and Cdc20KD cells were lysed and whole-cell lysates were pull-down for SMAR1 and immunoprecipitates were immunoblotted for K48-linked ubiquitin. (**e**) Cdc20 degrades SMAR1 through APC/C complex. NS and APC2KD cells were transfected either with vector control or HA-Cdc20 and protein lysates were immunoblotted for indicated proteins

To understand whether cellular levels of SMAR1 are regulated by Cdc20, we made Cdc20 stable knockdown MCF7 cells (Cdc20KD) by using two independent unrelated shRNAs (sh-Cdc20). Scramble shRNA knockdown cells were used as control (NS). As compared with NS cells, SMAR1 was found to significantly stabilize in Cdc20KD cells (Figure 4a). However, real time RT-PCR data suggest that Cdc20 does not affect the mRNA levels of *SMAR1* (Figure 4b). Next we investigated the half-life of SMAR1 in Cdc20KD cells using the cycloheximide chase assay, which revealed that the proteasomal turnover of SMAR1 is significantly suppressed in Cdc20KD cells, as compared with NS cells (Figure 4c). We also assessed K48-linked ubiquitylated levels of SMAR1 in Cdc20KD cells and found the significant reduction in Cdc20KD cells,as compared with NS cells. These data suggest that Cdc20 may be involved in the maintenance of cellular levels of SMAR1 through proteasomal degradation (Figure 4d). We then evaluated whether Cdc20 regulates SMAR1 levels in higher grades of breast cancer cell lines. To asses this, we depleted Cdc20 in MDA-MB-231 (TNBC) and T47D (Luminal A) cell lines and found the significant stabilization of SMAR1 without altering mRNA levels (Supplementary Figures 4a and b). Collectively results demonstrated that Cdc20 limits the expression of SMAR1 at the proteasomal levels in different breast cancer cell lines.

Cdc20 functions as one of the important substrate receptors of fourteen member APC/C complex. Cdc20 assembles with the APC/C complex through interaction with APC2. To understand whether Cdc20 regulates SMAR1 levels through the APC/C complex, we generated stable APC2 knockdown (APC2KD) cells using two unrelated shRNAs. Immunoblotting data revealed that SMAR1 levels were significantly stabilized in APC2KD cells, suggesting that SMAR1 is regulated through APC/C complex (Figure 4e). To further confirm this hypothesis, Cdc20 was ectopically expressed in NS and APC2KD cells and immunoblot showed that Cdc20 could degrade SMAR1 only in NS cells (Figure 4e). Next, endogenous K48-linked SMAR1 levels were examined in APC2KD cells. We found relatively lower levels of K48-linked SMAR1 in APC2KD cells than in NS cells (Supplementary Figure 4c). These results collectively suggest that Cdc20-mediated SMAR1 proteasomal degradation occurs through the APC/C complex.

### JNK kinase controls Cdc20-mediated degradation of SMAR1

**Figure 5:**
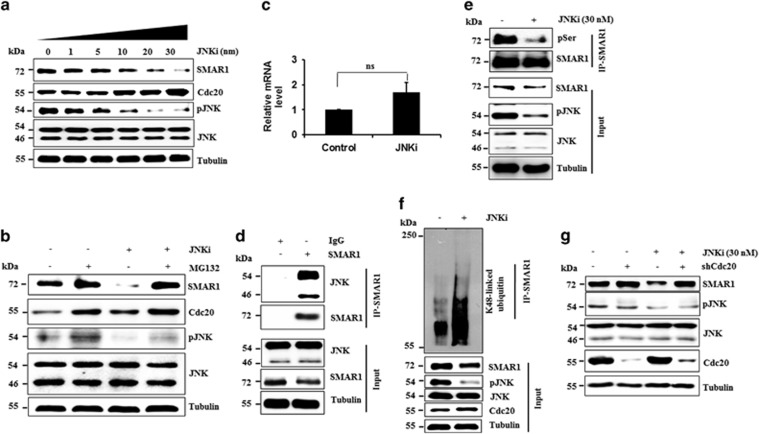
JNK prevents Cdc20-mediated degradation of SMAR1. (**a**) JNK regulates the stability of SMAR1. MCF7 cells were treated with increasing concentrations of JNK inhibitor for 12 h. Cell lysates were immunoblotted for indicated proteins. (**b**) JNK prevents proteasomal degradation of SMAR1. MCF7 cells were either untreated or treated with 30 nM JNK inhibitor in the absence and presence of 10 *μ*M MG132. JNK treatment was for 12 h and MG132 was for last 6 h. Cell lysates were immunoblotted for indicated proteins. (**c**) Relative mRNA level of SMAR1 upon JNK inhibition.SMAR1 mRNA levels were measured using RT-qPCR assay and normalized against GAPDH mRNA. NS represents not significant difference by one way ANOVA test. (**d**) SMAR1 interacts with JNK. Endogenous SMAR1 was immunoprecipitated and immunoprecipitates were immunoblotted with indicated antibodies. (**e**) JNK inhibition leads to reduced level of phospho serine of SMAR1. Endogenous SMAR1 was immunoprecipitated and immunoprecipitates were immunoblotted with indicated antibodies. (**f**) JNK inhibition leads to enhancement of K48-linked polyubiquitylation of SMAR1. MCF7 cells were treated with or without 30 nM JNK inhibitor and cell lysates were immunoprecipitated for SMAR1.Immunoprecipitates were immunoblotted with K48-linkage specific ubiquitin antibody. (**g**) Cdc20 degrades SMAR1 upon JNK inhibition. NS and Cdcd20KD cells were treated with JNK inhibitor, cells were lysed and immunoblotted for indicated proteins

Generally, APC/C^Cdc20^ recognizes phosphorylated substrates as targets for proteasomal degradation. Therefore, we checked the role of growth-associated kinases (ATM, MEK, JNK, AKT and mTOR) on SMAR1 levels using chemical inhibitors. Interestingly, JNK inhibition led to a significant decrease in the expression of SMAR1 in a dose-dependent manner (Figure 5a). SMAR1 levels were suppressed with a concomitant increase in Cdc20 stability, following JNK inhibition (Figure 5a). Further study revealed that JNK-mediated degradation of SMAR1 is a proteasomal process (Figures 5b and c). To further investigate JNK-mediated SMAR1 regulation, we performed co-immunoprecipitation assay and JNK was found to interacts with SMAR1 (Figure 5d). These observations prompted us to check whether JNK phosphorylates SMAR1. Our results demonstrated that levels of phosphorylation of serine residues of SMAR1 significantly decreased upon inhibition of JNK, indicating that JNK phosphorylates serine residues (Figure 5e). We then checked K48-linked ubiquitylation levels of SMAR1 in the presence of a JNK inhibitor and found the presence of a substantially high mass ladder of SMAR1 upon JNK inhibition. This further confirmed that JNK protects SMAR1 from proteasomal degradation (Figure 5f).

Figure 5a shows that JNK inactivation reveals a reciprocal relationship between SMAR1 and Cdc20 expression, indicating that Cdc20 might be involved in the degradation of SMAR1 following JNK inhibition. We therefore checked the levels of SMAR1 in Cdc20KD and NS cells in the absence and presence of JNK inhibitor and found that degradation of SMAR1 upon JNK inhibition was greatly inhibited in Cdc20KD cells, suggesting the involvement of Cdc20 (Figure 5g) in this degradation. Interestingly, the interaction of SMAR1 with Cdc20 was found to be increased upon inhibition of JNK (Supplementary Figure 5). These results collectively demonstrate that JNK is an important kinase responsible for the maintenance of cellular levels of SMAR1.

### Cdc20 fails to regulate SMAR1 under stress conditions

**Figure 6:**
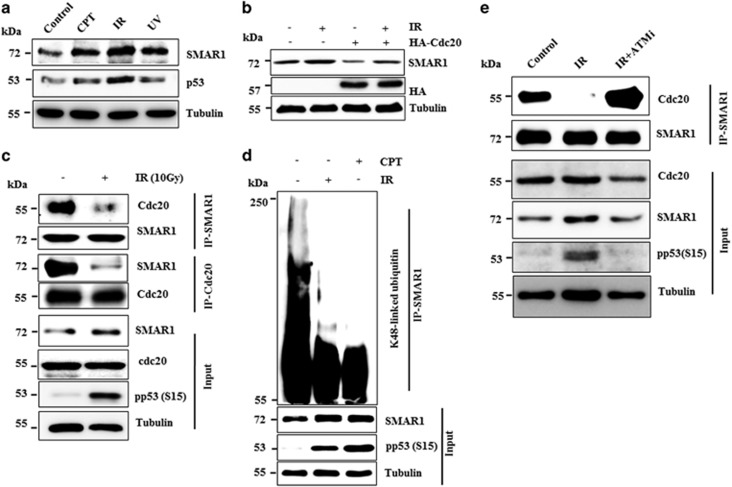
Genotoxic stress-mediated perturbation of SMAR1–Cdc20 interaction leads to SMAR1 stabilization. (**a**) SMAR1 isstabilized upon genotoxic stresses. MCF7 cells were treated with either 5 *μ*M CPT (Camptothecin) or IR (10 Gy), UV (10 J/m^2^) for 4 h. Cells were harvested, lysed and whole cell lysates were immunoblotted with indicated antibodies. (**b**) Cdc20 fails to degrade SMAR1 upon IR treatment. MCF7 cells were transfected either with vector or Cdc20 for 36 h. Transfected cells were then treated with and without IR for 4 h. Cells were collected, lysed and analyzed by western blotting using indicated antibodies. (**c**) Cdc20 fails to interact with SMAR1 upon DNA damage. Irradiated and un-irradiated MCF7 cell lysates were immunoprecipitated either with SMAR1 or Cdc20 antibody. Immunoprecipitates were analyzed by immunoblotting with indicated antibodies. (**d**) K48-linked polyubiquitylation level of SMAR1 was suppressed upon DNA damage. MCF7 cells were either untreated or treated with 5 *μ*M CPT or 10 Gy IR for 4 h. Cell lysates were prepared and used for immunoprecipitation with anti-SMAR1 antibody. Immunoprecipitates were analyzed by immunoblotting using K48-linked ubiquitin antibody. (**e**) ATM regulates the interaction of SMAR1 and Cdc20.MCF7 cells were either untreated or irradiated with 10 Gy IR in the absence and presence of ATM inhibitor (KU55933). Cell lysates were prepared and immunoprecipitated with anti-SMAR1 antibody and analyzed by immunoblotting with indicated antibodies

Previous reports have shown that SMAR1 has an important role in DNA damage repair and apoptosis upon exposure to genotoxic stress.^5, 6^ In concurrence with these reports, we observed that SMAR1 is significantly stabilized upon treatment with camptothecin (CPT), ionizing radiation (IR) and UV irradiation (Figure 6a). We then examined the role of Cdc20 in SMAR1 stability under genotoxic stress. As seen in Figure 6b, proteasomal degradation of SMAR1 by Cdc20 is significantly inhibited upon exposure to IR, suggesting that Cdc20 may be unable to interact with and/or polyubiquitinate SMAR1 under genotoxic stress. Indeed, coimmunoprecipitation results revealed that the interaction of SMAR1 with Cdc20 is significantly perturbed upon IR treatment (Figure 6c) and therefore may affect polyubiquitylation levels of SMAR1. Results revealed that K48-linked polyubiquitylation levels of SMAR1 were notably reduced upon exposure to genotoxic stresses, further confirming that interaction between SMAR1 and Cdc20 have a crucial role in SMAR1 stabilization (Figure 6d).

It has been recently shown that ATM phosphorylates SMAR1 at the serine 370 residue, in response to genotoxic stress.^6^ So we investigated whether ATM-mediated phosphorylation of SMAR1 has any role in interactions between SMAR1 and Cdc20 upon genotoxic stress. Our results demonstrated that significant perturbation of SMAR1–Cdc20 upon ionizing radiation was restored following inhibition of ATM (Figure 6e). These observations suggest that ATM-dependent phosphorylation of SMAR1 at Ser 370 may be responsible for the loss of SMAR1–Cdc20 interactions, which intern leads to the stabilization of SMAR1, allowing it to perform its repair functions.

### Cdc20 limits the SMAR1 tumor suppressive activity in higher grades of breast cancer

**Figure 7:**
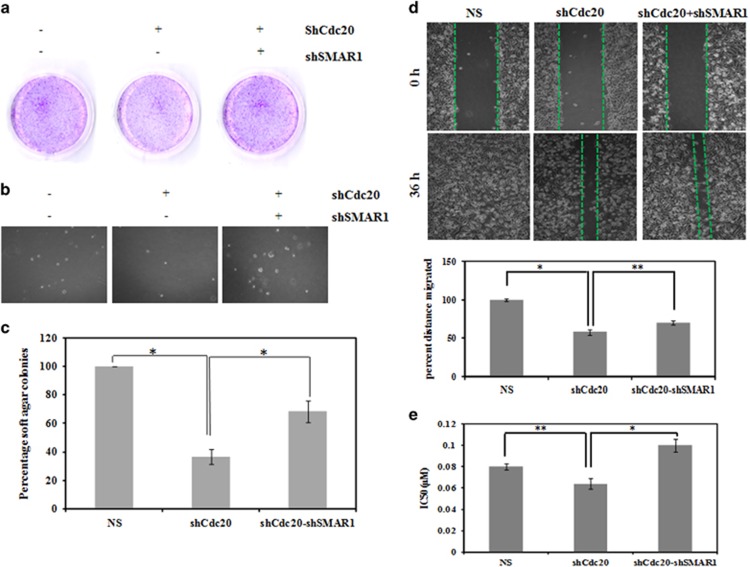
Cdc20 impairs the tumor suppressive function of SMAR1 in higher grade of breast cancer. (**a**) Depletion of Cdc20 suppresses growth of MDA-MD-231. Long term survival assay (Colony formation assay) showed the colony formation ability of MDA-MB-231 cells expressing either scramble shRNA or shCdc20 or coexpression of both shCdc20 and shSMAR1. (**b**) Soft agar analysis assay showed the anchorage independent growth potential of MDA-MB-231 cells expressing either scramble shRNA or shCdc20 or coexpression of both shCdc20 and shSMAR1. (**c**) Quantification of colonies from soft agar assay. An *asterisk* indicates the significant difference (*P<0.05) calculated by one way ANOVA test. (**d**) Cell migration potential of MDA-MB-231 cells expressing either scramble shRNA or shCdc20 or coexpression of shCdc20 and shSMAR1. Quantification of migration data were presented graphically in the lower panel. An *asterisk* indicates the significant difference (*P<0.05) calculated by one way ANOVA test. (**e**) Graphical representation of IC50 value for doxorubicin in MDA-MB-231 cells upon stable knockdown of Cdc20 and Cdc20 and SMAR1. An *asterisk* indicates the significant difference (*P<0.05) calculated by one-way ANOVA test

To investigate the biological relevance of the SMAR1–Cdc20 reciprocal relationship, we performed the long-term survival assay and anchorage-independent growth assay using MDA-MB-231 cells stably expressing NS or shRNA for Cdc20 or shRNA for both Cdc20 and SMAR1. Immunoblots showed the SMAR1 and Cdc20 levels in respective depleted cells (Supplementary Figure 6a). Long-term survival showed that depletion of Cdc20 resulted in less number of colonies, while the number of colonies was more when SMAR1 and Cdc20 were co-depleted (Figure 7a). Similar results were also observed in an anchorage-independent soft agar assay (Figures 7b and c). We then assessed the cell migration potential (scratch wound healing) of these stable knockdown cells. Our results revealed that Cdc20 depletion led to retardation of cell migration, while cells co-depleted for Cdc20 and SMAR1 migrated to a better extent (Figure 7d). These observations show that Cdc20 promotes tumorigenesis by inhibiting the SMAR1 tumor suppressor activity. To gain further insights, SMAR1-WT and SMAR1-D1 (defective in proteasomal degradation) were overexpressed in the absence and presence of Cdc20 and the scratch wound cell migration assay was performed. The migration of cells expressing either SMAR1-WT or SMAR1-D1 was significantly inhibited (Supplementary Figure 6b). However, coexpression of Cdc20 in SMAR1-WT-expressing cells led to an increase in cell migration (Supplementary Figure 6b). On the other hand, Cdc20 coexpression failed to alter the cell migration of SMAR1-D1-expressing cells (Supplementary Figure 6b). Collectively, our results suggest that Cdc20 augments cell migration of cancer cells by proteasomal degradation of SMAR1, at least in part.

We next investigated the invasiveness of breast cancer cells expressing either SMAR1-WT or SMAR1-D1 in the absence and presence of ectopic expression of Cdc20 (Supplementary Figure 6c). The invasiveness of breast cancer cells expressing either SMAR1-WT or SMAR1-D1 were found to be substantially inhibited. Interestingly, coexpression of Cdc20 in cells expressing SMAR1-WT resulted in increased invasion. In contrast, it had no effect on cells expressing SMAR1-D1. Thus, our results demonstrate that Cdc20 augments cancer cell invasion through proteasomal degradation of SMAR1, at least in part.

Previous study has shown that the tumor suppressor SMAR1 induces apoptosis upon genotoxic stress.^5^ So we measured the chemo sensitivity (inhibitory concentration 50, IC_50_) of stable Cdc20 knockdown MDA-MB-231 cells using doxorubicin. The results showed that knockdown of Cdc20 leads to higher sensitivity (IC_50_ – 0.064±0.005 *μ*M) to doxorubicin, as compared with control NS cells (IC_50_ – 0.08± 0.003 *μ*M). The higher sensitivity of Cdc20-depleted cells may be due to increased levels of SMAR1. To test this possibility, we knocked down SMAR1 in Cdc20-depleted cells. The results revealed that the sensitivity of Cdc20-depleted cells (IC_50_ – 0.064± 0.005 *μ*M) towards doxorubicin decreased upon co-depletion of SMAR1 (IC_50_ – 1.04±0.006 *μ*M). These observations are in accordance with the pro-apoptotic and anti-apoptotic roles of SMAR1 and Cdc20, respectively (Figure 7e). Collectively, our results demonstrate that Cdc20 impairs the tumor suppressive function of SMAR1 and thereby increases malignancy, at least in part.

### The reciprocal relationship of SMAR1 and Cdc20 in breast cancer

**Figure 8:**
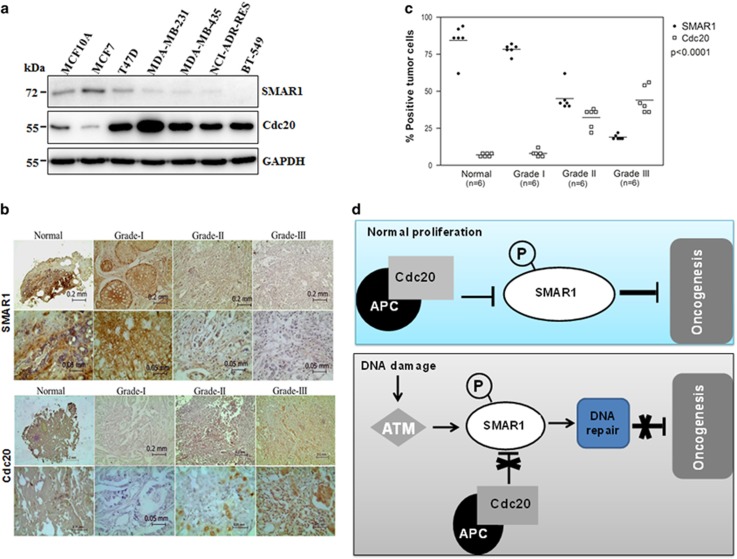
Reciprocal relationship of Cdc20 and SMAR1 in breast cancer. (**a**) Expression of SMAR1 shares an inverse correlation with Cdc20 in higher grades of breast cancer cell lines. (MCF 10A: Primary breast epithelial cell line, MDA-MB-435: Basal B and BT 549: Basal B tripe negative breast cancer cell line, rest of the cell line information given in Supplementary Figure 1a).^22, 23, 24^ (**b**) Expression level of SMAR1 (upper panel) and Cdc20 (lower panel) in normal and higher grades of breast cancer patient samples. Tissues were stained for immunohistochemical analysis as described in the Materials and Methods. (**c**) Statistical analysis of the average score of SMAR1 and Cdc20 staining between cancer tissues and corresponding non-tumor tissues, *P*<0.0001 (one-way ANOVA). (**d**) Model depicting the regulation of SMAR1 in normal and genotoxic stress conditions by APC/C^Cdc20^

To further gain insights into the converse relationship of SMAR1 and Cdc20, we examined the expression of SMAR1 and Cdc20 in a panel of breast cancer cell lines.^22, 23, 24^ Immunoblotting data showed that the levels of expression of Cdc20 are significantly increased with a concomitant decrease in levels of SMAR1 in metastatic breast cancer cell lines (Figure 8a), which is in concurrence with the previous reports.^8, 21^ We performed IHC of samples from different grades of breast cancer patient to examine the expression of SMAR1 and Cdc20 and found the converse relation of SMAR1 and Cdc20 (Figures 8b and c). The levels of expression of SMAR1 were significantly repressed while those of Cdc20 were significantly elevated with increasing grades of cancer further supporting the *in vitro* cell lines results. Thus our findings have elucidated that Cdc20-mediated proteasomal degradation of SMAR1 limits its tumor suppressive activity.

## Discussion

The tumor suppressor SMAR1, has an important role in cell cycle progression and apoptosis, is reported to be under-expressed in higher grades of cancer.^3, 4, 5, 6, 8^ Interestingly, SMAR1 is reported to be stabilized under genotoxic stress to help DNA damage repair through the recruitment of Ku-70/80.^6^ However, the underlying molecular mechanism of its repression in higher grades of cancer and the genotoxic stress-mediated stabilization was not clearly understood. This prompted us to investigate how cellular levels of SMAR1 are regulated under normal and genotoxic stress. We observed a substantial post-translational (proteasomal) regulation of SMAR1 in different breast cancer cell lines (Supplementary Figure 1a). Therefore, we performed Ring-finger E3 ligase (SCF and APC/C complex) screens, which led to the identification 4 F-box proteins and Cdc20 to be involved in SMAR1 stability (Figure 1c).

Studies suggest that Cdc20 is frequently overexpressed in many of the higher grades of cancer.^16, 17, 25, 26, 27^ Moreover, selective depletion of Cdc20 leads to the inhibition of tumor growth and metastasis.^28^ Previous reports revealed that SMAR1 expression was ablated in higher grades of breast cancer.^4^ Therefore, the elevated levels of Cdc20 in higher grades of cancer could be associated with lower SMAR1 expression. In addition, the presence of D-box motifs in SMAR1 prompted us to select the *Cdc20* gene for further study. We observed that Cdc20 binds with and directs K48-linked polyubiquitylation-mediated proteasomal degradation of SMAR1.

We observed that SMAR1 and Cdc20 shared reciprocal levels of expression in higher grades of breast cancer cell lines and patient samples (Figures 8a and b). The converse relationship is supported by the half-life of SMAR1 in MDA-MB-231 and MCF7 cell line (Figure 2f and Supplementary Figure 2) and the abundance of the K48-linked polyubiquitylated ladder of SMAR1 (Figure 2g). Our study provided new insights into the mechanisms of the oncogenic role of Cdc20 in promoting cell invasion, migration, anchorage-independent growth, chemosensitivity, by suppressing the expression of the tumor suppressor SMAR1.

Previous studies have shown that activation of JNK leads to the suppression of cell growth and induction of apoptosis. Our data have demonstrated, for the first time, that JNK may prevent malignancy through stabilizing the SMAR1. Our data revealed that JNK inhibition led to stronger interactions of SMAR1 with Cdc20, indicating that JNK-mediated phosphorylation of SMAR1 prevents Cdc20-mediated proteasomal degradation (Supplementary Figure 5). It is also possible that phosphorylation of SMAR1 leads to its conformational change, which is less accessible to Cdc20 for proteasomal degradation. Thus, SMAR1-mediated activation of cell cycle arrest, DNA damage response and apoptosis may partly due to JNK-mediated protection of SMAR1.

SMAR1 is stabilized and regulates apoptosis (through Bax and PUMA) and DNA repair by regulating Ku-70/80 and HDAC6 associations upon genotoxic stress.^5, 6^ However, the molecular mechanism of SMAR1 stabilization upon genotoxic stresses was not well understood. Previous report showed that ATM phosphorylates SMAR1 at Ser-370 following DNA damage.^6^ In this study, we show that Cdc20 fails to interact with and degrades SMAR1 in ATM dependent manner following genotoxic stress.

The findings of this study have helped in elucidating for the first time, the intriguing molecular mechanism of regulation of SMAR1 expression by oncogenic Cdc20 in unstressed cells as well as in cells subjected to genotoxic stress (Figure 8d). We found that Cdc20 functions as an oncogene by limiting the tumor suppressive activity of SMAR1 in higher grades of breast cancer, at least in part. Small molecules could be designed to disrupt the interactions between Cdc20 and SMAR1, to help restore the tumor suppressive function of SMAR1, leading to improved cancer therapy in the future.

## Materials and Methods

### Cell culture and tissue samples

Human MCF10A, MCF7, MDA-MB-231, NCI-ADI-RES, MDA-MB-435, BT-549, Hela and HCT-116 cancer cell lines and transformed human embryonic kidney (HEK293T) cells were kind gift from Prof. Michael R. Green (University of Massachusetts Medical School, USA). MCF7, BT-549, MDA-MB-435, Hela and HEK 293T cells were cultured at 37 °C in Dulbecco’s modified Eagles medium (DMEM) (Gibco, Life Technologies, Carlsbad, CA, USA) containing 10% fetal bovine serum (FBS) (Gibco). MCF 10A cells were cultured in DMEM F12 (Gibco) with 10% horse serum. Human breast cancer cell lines MDA-MB-231, NCI-ADI-RES and HCT116 were maintained in Roswell Park Memorial Institute medium (RPMI 1640) with 10% FBS supplement. All tissue culture media were supplemented with 2 mM l-glutamine, 25 *μ*g/ml streptomycin and 25 U penicillin (Gibco). Cells were cultured in a humidified atmosphere with 5% CO2 at 37 °C.

Paired breast cancer tissues and adjacent non-tumor breast tissues were collected from therapeutic surgery. All samples were obtained with informed consent.

### Plasmid transfection

The mammalian expression constructs for expression of human F-box genes (55 cDNA clones of F-box genes), pCMV-FLAG-SMAR1, pCMV-HA-Cdc20^29^ (Addgene, Cambridge, MA, USA) were used in this study. All the F-box cDNA clones were cloned in pCMV-Entry 6 myc/DDk (Origene). Transient transfections were performed by using Lipofectamine 2000 (Invitrogen) according to the manufacturer’s instructions.

### RING finger E3 ubiquitin ligase screen

RING-finger E3 ubiquitin ligase screen was performed in MCF7 cells. F-box genes (55 F-box genes), and anaphase promoting complex adapter proteins Cdh1 and Cdc20 were ectopically overexpressed in MCF7 cells for 48 h. Cells were then harvested, lysed and processed for immunobloting. Three independent screens were performed and candidates showing maximum degradation of SMAR1 were selected and again transfected for the 4th time to validate the candidates.

### Treatment of cells

Cells were treated with either vehicle or 10 *μ*M MG132 (Calbiochem, USA) or 30 nM JNK inhibitor (Calbiochem, USA) or 40 *μ*g/ml cycloheximide (Sigma, USA) for the indicated time points. The cells were harvested after treatment and whole-cell extracts were prepared.

### Cell lysate preparation and immunoblotting

Cells were harvested and washed twice with ice cold phosphate buffer saline (PBS). Cells were then lysed with whole cell lysis buffer (50 mM Tris pH7.4, 200 mM NaCl, 50 mM NaF, 1 mM Na_3_VO_4_, 0.5% Triton X-100 and protease inhibitor cocktail) in ice for 30 min.^30^ Lysates were centrifuged at high speed (16 000 × *g*) and clear supernatants were transferred to new tubes. Protein concentration was measured by the Bradford method using bovine serum albumin as a standard.^31^ Samples were prepared in SDS sample buffer and run in SDS-PAGE with Tris-Glycine (25 mM Tris, 192 mM Glycine) running buffer containing 0.1% SDS. Separated proteins were transferred onto PVDF membrane with transfer buffer (80% Tris-Glycine and 20% Methanol). Membranes were incubated with primary antibody for overnight at 4 °C and were subsequently incubated with HRP conjugated secondary antibody for 1 h at ambient temperature. Blots were developed by the chemiluminescence method. Densitometry analysis of the immunoblots was performed using the image J software.

The antibody against SMAR1 was obtained from Bethyl (USA). *β*-actin and *α*-tubulin were obtained from Sigma. JNK, phospho JNK (Thr183/Tyr185), APC2, total ERK, were obtained from Santa Cruz Biotechnology (Santacruz, CA, USa). *β*catenin, LC3b and phospho AKT (serine 473), Phospho S6K, total AKT, total S6K, phospho ERK (Thr 302/Tyr 304), phpspho p53 (serine 15) and K48-linked ubiquitin antibodies were obtained from Cell signaling technology, USA. The DDK antibody was purchased from Origene, USA.

### Cycloheximide pulse chase experiments

Cycloheximide was purchased from Sigma. It was freshly prepared in 25 mM PIPES buffer pH 5.8 for treatment. Cells were pulse chased with 40 *μ*g/ml cycloheximide for the indicated time points. The cells were then harvested and lysed as described in the Cell lysate preparation and immunoblotting sections.

### Fractionation of nuclear and cytoplasmic proteins

Cells were harvested and washed with ice cold PBS followed by centrifugation at 4200 × *g* for 2 min at 4 °C. The cell pellet was then resuspended in 200 *μ*l of hypotonic buffer (10 mM HEPES-K^+^ pH 7.5, 10 mM KCl, 1.5 mM MgCl_2_, 0.1 mM DTT and 0.5% Triton X-100) containing protease inhibitor cocktail and incubated in ice for 5 min. The cell suspension was then centrifuged at 4200 × *g* for 2 min at 4 °C. The supernatant was collected in a separate tube (cytoplasmic fraction) followed by washing of the cell pellet with cold wash buffer (10 mM HEPES-K^+^ pH 7.5, 10 mM KCl, 1.5 mM MgCl_2_, 0.1 mM DTT). The washed pellet was lysed in lysis buffer (50 mM Tris pH7.4, 200 mM NaCl, 50 mM NaF, 1 mM Na_3_VO_4_, 0.5% Triton X-100 and protease inhibitor cocktail) for 30 min in ice. The lysate was then centrifuged at 16 000 × *g* for 30 min and the supernatant was collected as the nuclear fraction.

### Immunoprecipitation

Cells were lysed in cell lysis buffer (50 mM Tris pH7.4, 200 mM NaCl, 50 mM NaF, 1 mM Na_3_VO_4_, 0.5% Triton X-100 and protease inhibitor cocktail). Whole-cell lysate (600–800 *μ*g of proteins) was used for co-immunoprecipitation using 2 *μ*g of antibody in modified IP lysis buffer (50 mM Tris pH7.4, 200 mM NaCl, 50 mM NaF, 1 mM Na_3_VO_4_, 0.1% Triton X-100 and protease inhibitor cocktail). The mixture of protein and antibody was kept at 4 °C in a rotor with gentle rocking for 12–16 h. The following day this antibody and cell lysate cocktail was allowed to bind to protein G-agarose beads for 2 h at 4 °C with gentle rocking. The immunoprecipitates were eluted from the beads using Laemmli buffer for 3–5 min and boiled prior to resolving on SDS-PAGE. Three percent of the proteins taken in immunoprecipitation experiment were used as input in all immunoprecipitation assays.

### Lentivirus generation for stable knockdown cells

Lentiviral pGIPZ shRNAs against all the genes were a kind gift from Prof. Michael R. Green, University of Massachusetts Medical School, USA. shRNAs along with packaging vectors were co-transfected in HEK293T cells using polyethyleneamine (Polyscience, USA). Media supernatant were collected after 48 h of transfection, and passed through 0.45 *μ*m syringe filters to collect the virus-containing supernatant. Cells were infected with the virus soup in the presence of 8 *μ*g/ml polybrene and the infected cells were selected by puromycin selection. A non-target shRNA (Scramble shRNA) against the human genome was used as a control.

### Colony formation assay

For colony formation assay, 5000 infected cells were seeded in a 35-mm culture dish and allowed to grow for 12–15 days. Subsequently the cells were fixed with 3.7% formaldehyde and stained with 0.05% crystal violet. The plates were photographed and representative images were included under the result section.

### Soft agar assay

For the anchorage independent soft agar growth assay, 35-mm dishes were filled with 0.6% base agar (Invitrogen) and 2X RPMI 1640. Five thousand infected cells were then suspended in 0.03% of top agar containing 10% FBS and were placed on top of the base agar. Three weeks later, colonies were observed under the microscope and photographed.

### Determination of IC_50_ value of doxorubicin

The cell viability of control NS and knockdown cells in the presence of doxorubicin was measured using the MTT [3-(4,5-dimethylthiazol-2- yl)-2,5-diphenyltetrazolium bromide] assay as previously described.^32^

### Migration and invasion assay

For scratch wound healing cell migration assays, cells were seeded into 35 mm culture dishes and were allowed to grown into confluent monolayer. A scratch was made across the middle of each dish by using a 20 *μ*l pipet tip, followed by removal of cellular debris by washing with phosphate buffered saline (PBS). The cultures were incubated at 37 °C in a CO_2_ chamber and photographed. The relative area covered by the migrated cells was determined. Each sample was assayed in triplicate, and a minimum of three independent experiments were performed.

For the *in vitro* invasion assay, cells were serum starved after 24 h of transfection and 50,000 cells were seeded in the upper chamber of a transwell plate in 200 *μ*l of media containing 0.5% FBS. Medium with 10% FBS (1 ml) was added in the lower chamber. After incubation for 12 h at 37 °C in a CO_2_ chamber, the medium and the non migrated cells on the upper chamber were removed by cotton swab. The cells were then fixed with 3.7% formaldehyde, followed by washing with PBS and were stained with 0.5% crystal violet stain and photographed. Five fields were randomly captured and the number of migrating/invading cells was expressed as the average number of cells per microscopic field over five fields.

### Quantitative real time RT-PCR

Total RNA was prepared by using TRIzol reagent (Invitrogen) according to the manufacturer’s protocol. One microgram of RNA was reverse transcribed into cDNA using the BioRad cDNA preparation kit according to the manufacturer’s instructions. Real-time PCR (RT-PCR) was performed with the TAKARA superscript II SYBR green qPCR Mix. The expression of GAPDH was used to normalize the mRNA level of the gene of interest. Relative mRNA levels were determined considering untreated/zero time point as considering as 1. The primers used were as follows

GAPDH forward: AATCCCATCACCATCTTCCA

GAPDH reverse: TGGACTCCACGACGTACTCA

SMAR1 forward: GGTACCGAATCAAGCAGAGC

SMAR1 reverse: GCAGTAGGAGGACGAGTTGG

### Radiation treatment

For the ionizing radiation treatment, the cells were irradiated with a Co^60^ irradiator and were harvested 6 h post-irradiation and processed further for different experiments. Another set of cells were irradiated with 10 J/m^2^UV radiation and harvested after 4 h.

### Ubiquitination assay

MCF7 cells were co-transfected with different combination of FLAG-SMAR1, HA-Cdc20 and His-Ubiquitin to assess the *in vivo* ubiquitination of SMAR1. Cells were harvested after 48 h of transfection with the addition of 10 *μ*M MG132 6 h prior to harvesting the cells. Cells were harvested and lysed and 800 *μ*g of whole-cell lysate was immunoprecipitated using anti-FLAG antibody. The resultant immunoprecipitates were resolved in SDS-PAGE and probed for anti-His antibody to assess relative ubiquitylated levels of SMAR1 in the presence and absence of Cdc20. For ubiquitination assay under specific treatments, MCF7 cells were transfected either with the vector or HA-Cdc20 in the absence and presence of either JNK inhibitor or DNA damaging agents like gamma radiation or camptothecin (CPT) for the indicated time points. Cells were treated with MG132 for 6 h before harvesting and were then collected and lysed in cell lysis buffer. SMAR1 was immunoprecipitated with anti-SMAR1 antibody and separated by SDS-PAGE, followed by immunoblotting with anti-K48-linked ubiquitin antibody. To determine the effect of Cdc20 overexpression on SMAR1 ubiquitination, MCF7 cells were transfected with empty vector or HA-Cdc20 for 36 h,and then incubated with 10 *μ*M MG132 for an additional 6 h. Endogenous SMAR1 was immunoprecipitated from cell lysates and probed for ubiquitin K48-linked ubiquitin antibody. A similar strategy was followed to investigate SMAR1 endogenous ubiquitination upon JNK inhibition and under genotoxic stress. MCF7 cells were treated with 30 nM of JNK inhibitor or subjected to DNA damage with exposure to ionizing radiation (10 Gy) or treatment with 5 *μ*M of CPT for 6 h, and SMAR1 endogenous ubiquitination was then examined.

### Immunohistochemistry

The tissue samples were obtained from SDM College of Medical Sciences, according to established core procedures and approval from the Institutional Ethical Board. Tissue samples were stained with hematoxylin–eosin to determine the histological type and grade of tumors. SMAR1 and Cdc20 protein levels in the 18 tissue samples from breast cancer patients, including cancerous tissue and adjacent non-malignant epithelium, were detected using standard immunohistochemical staining procedure. In brief, after deparaffinization and endogenous peroxidase blockage, the sections were heated in a 0.01 M citrate buffer solution (pH 6.0) in a water bath at 98 °C for 20 min, then incubated with the antibody for SMAR1 and Cdc20 (Santacruz Biotechnology) at 1:100 dilution overnight at 4ºC, and visualized using the 3,3’-diaminobenzidine (DAB) detection kit (Vector labs). For the negative control, anti-rabbit IgG whole molecule (Sigma–Aldrich) was used at 1:1000 dilution. IHC stained samples were evaluated by two pathologists and all samples were blinded. The staining intensity of these proteins in neoplastic cells was graded on a scale of 0 (no staining) to 3+ (strong staining). The protein expression was scored based on the percentage of positive cells: 0=0% of stained positive cells; 1=weakly stained tissue or 1–25% of positive cells; 2=moderate stained tissue or 26–50% of positive stained cells; and 3=strongly stained tissue or more than 50% of stained cells.

### Statistical analysis

Each experiment was repeated at least three times. Values were shown are mean±S.E.M., except when mentioned otherwise.
