# Supplementary figures and images for: Cdc20 directs proteasome-mediated degradation of the tumor suppressor SMAR1 in higher grades of cancer through the anaphase promoting complex
Source: Cell Death Dis. 2017 Jun 15;8(6):e2882–. doi: 10.1038/cddis.2017.270 (PMC5520925; doi:10.1038/cddis.2017.270)

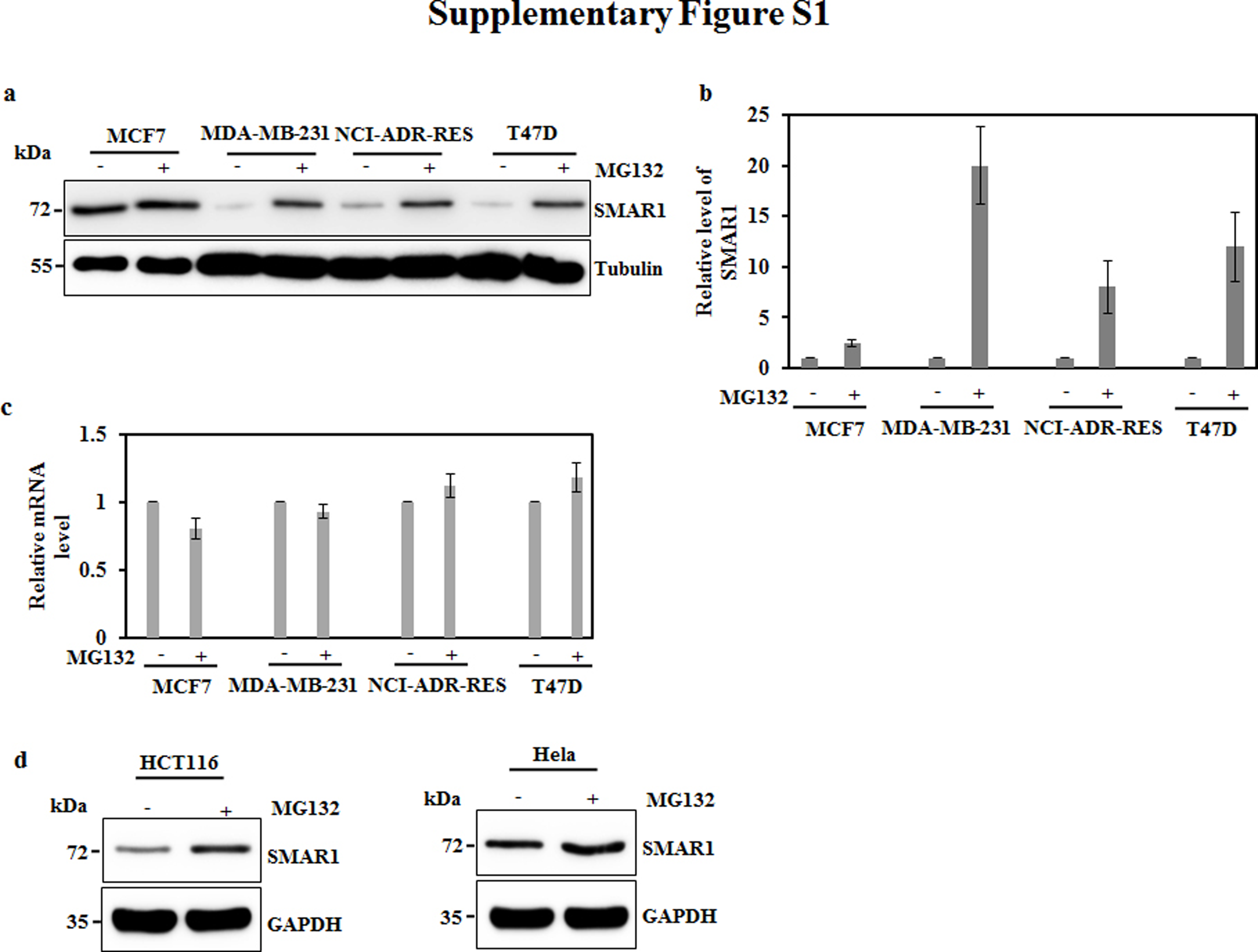

Supplement: Supplementary Figure S1 [file cddis2017270x1.tif]

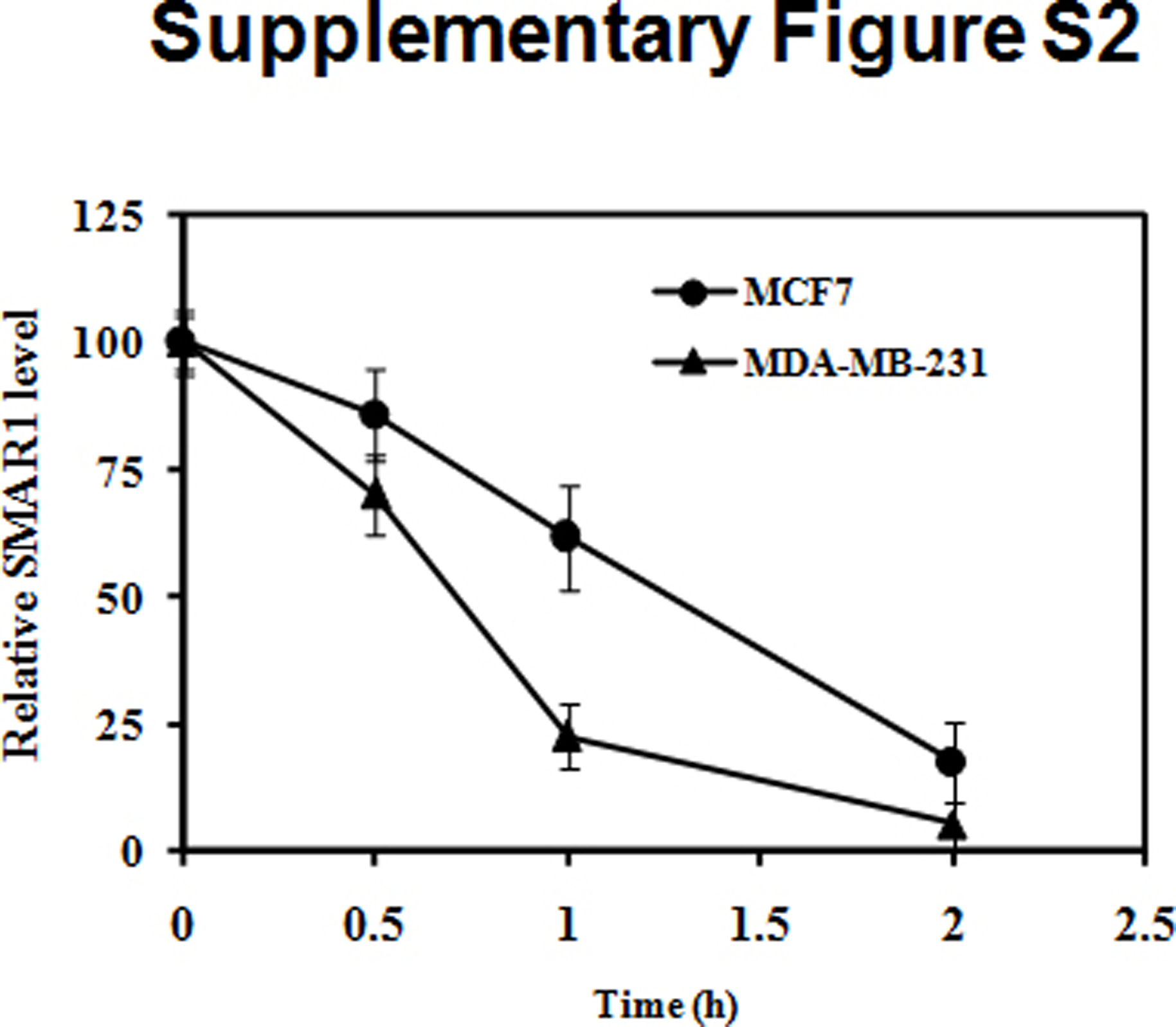

Supplement: Supplementary Figure S2 [file cddis2017270x2.tif]

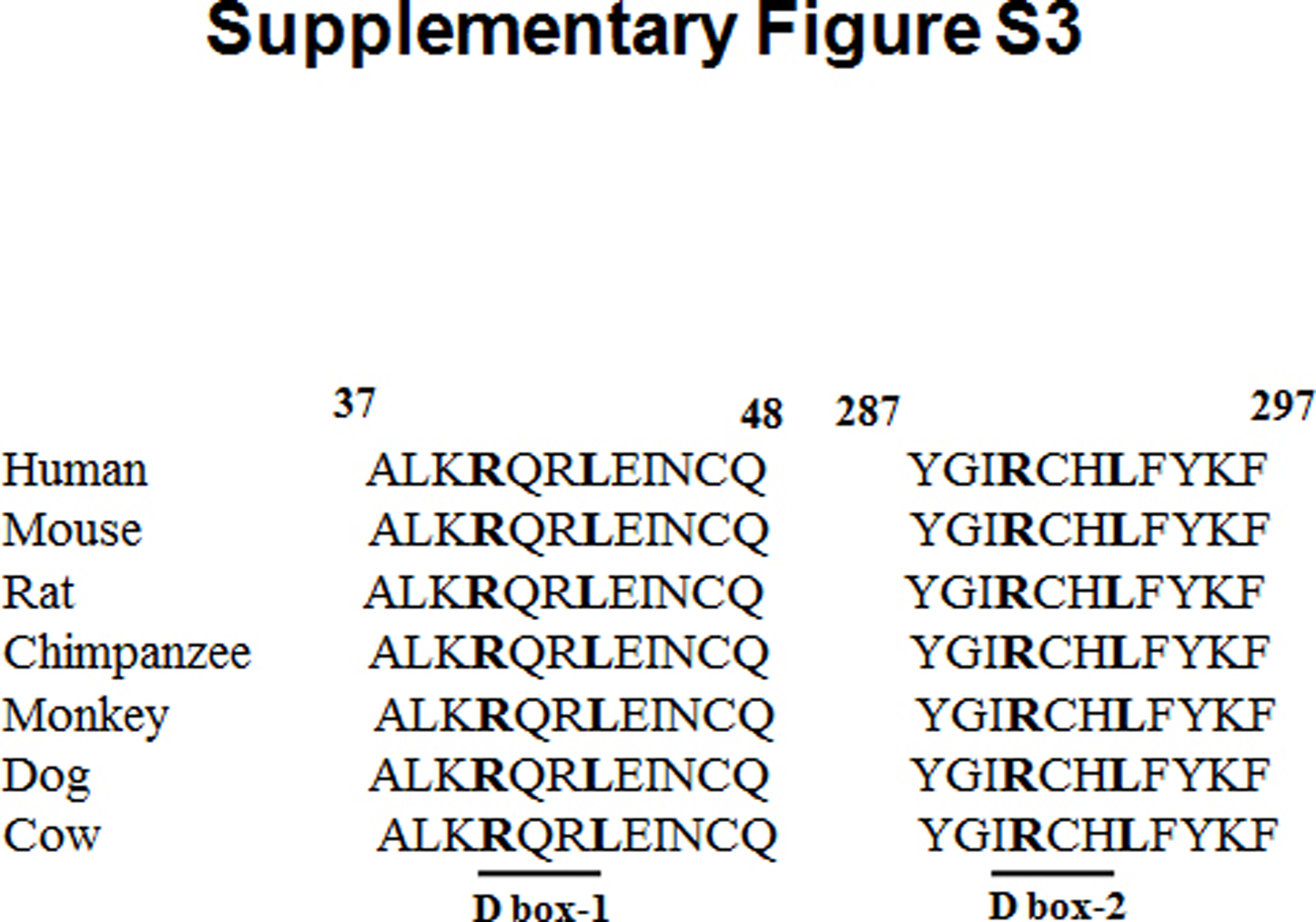

Supplement: Supplementary Figure S3 [file cddis2017270x3.tif]

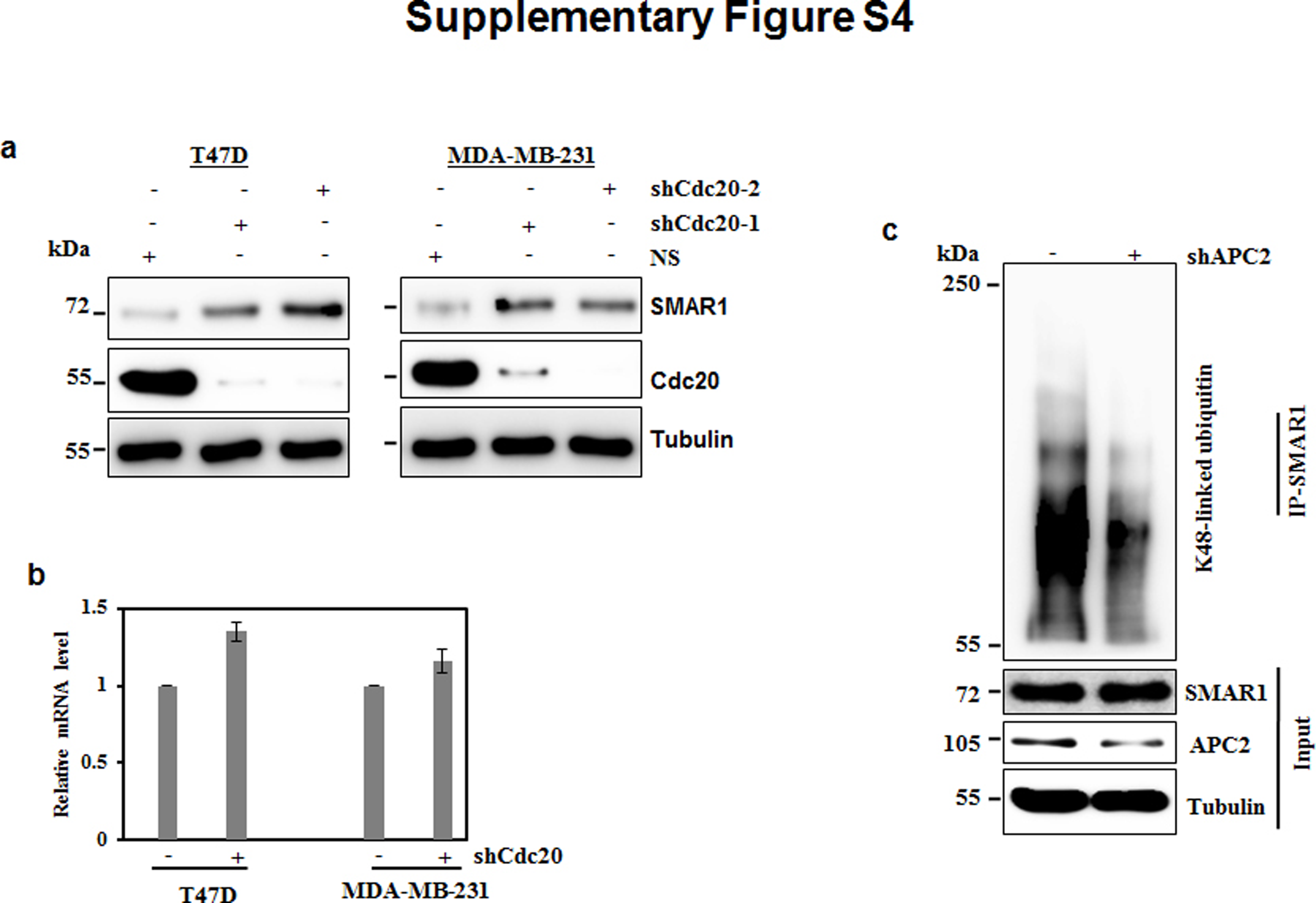

Supplement: Supplementary Figure S4 [file cddis2017270x4.tif]

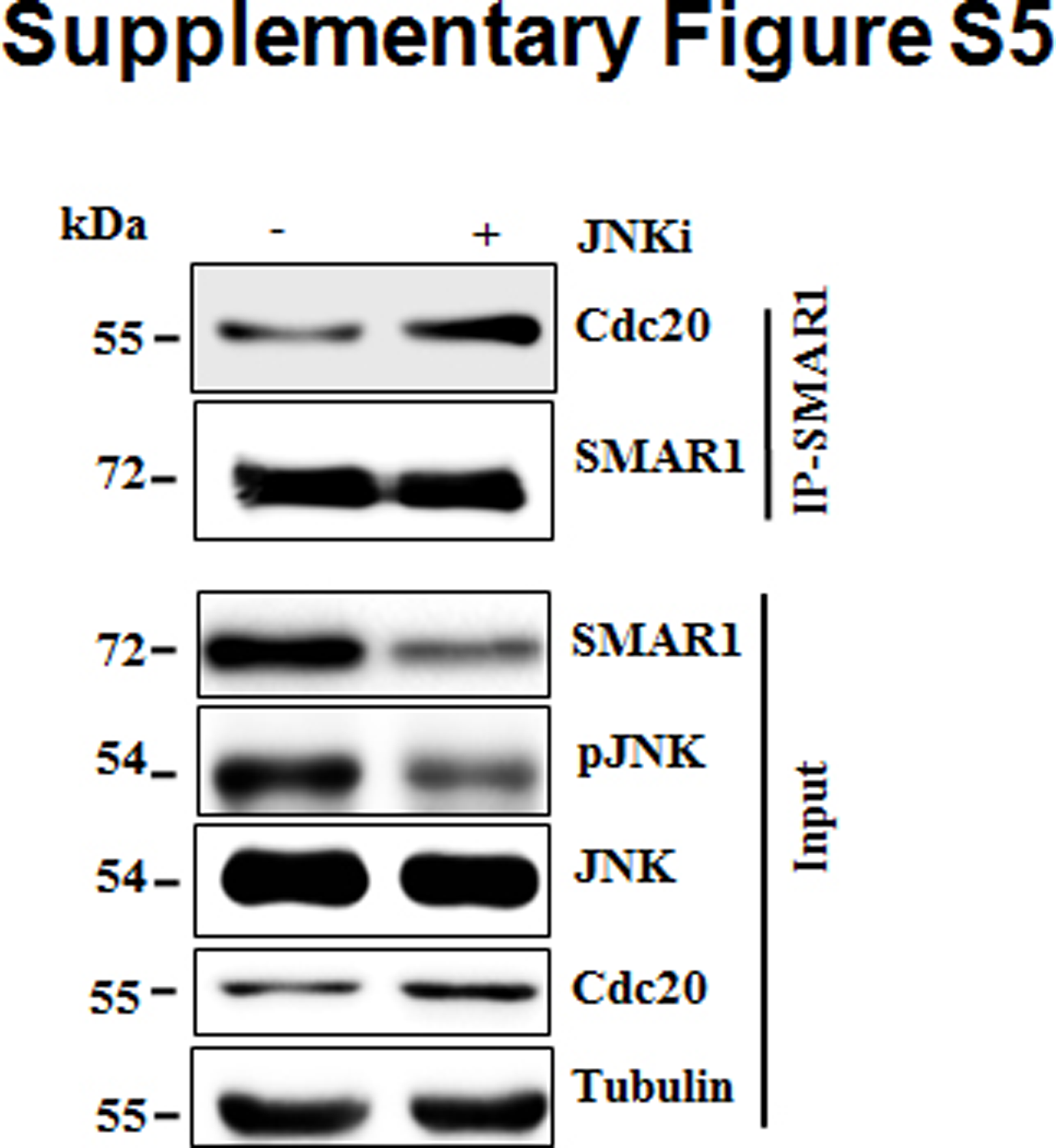

Supplement: Supplementary Figure S5 [file cddis2017270x5.tif]

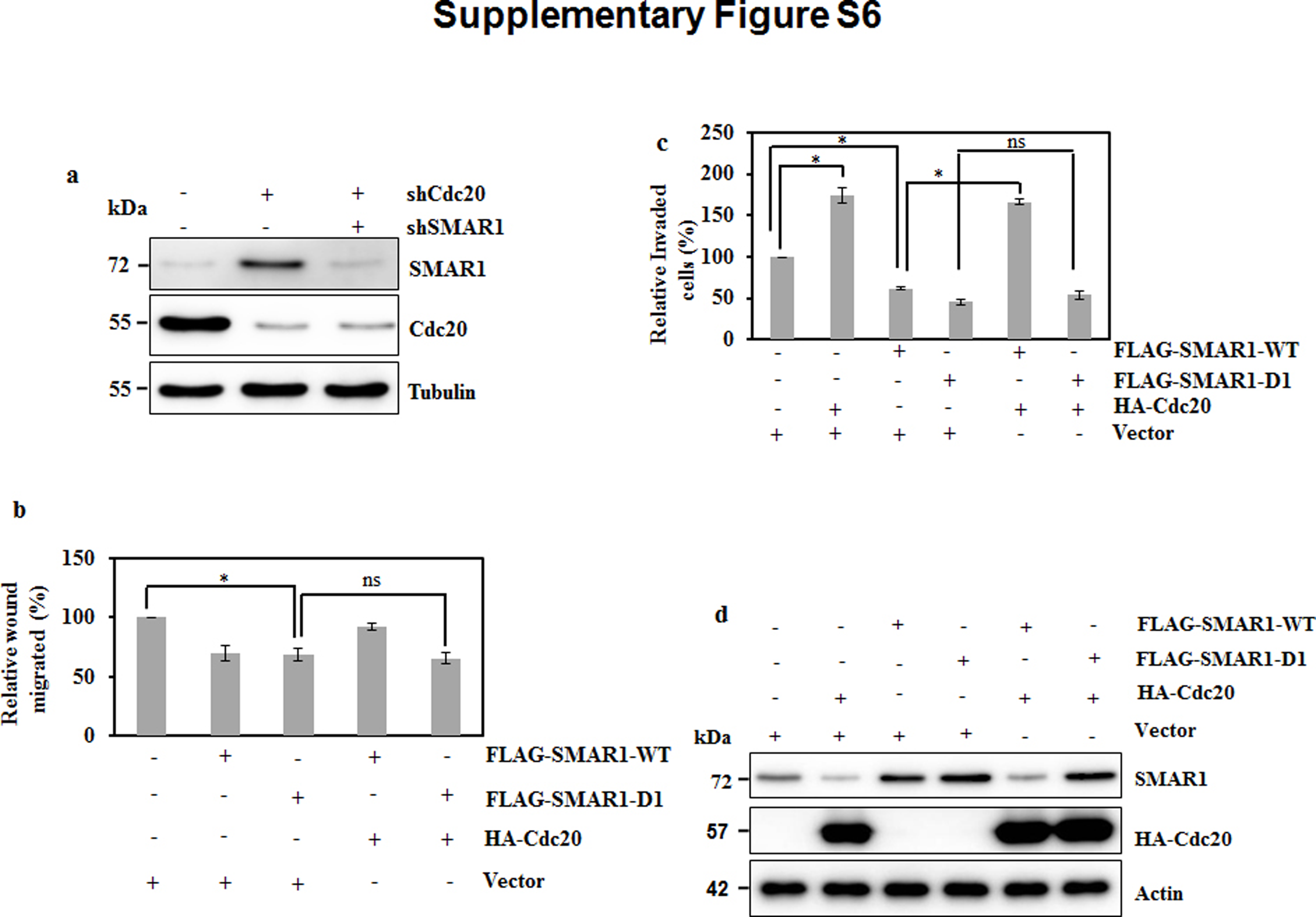

Supplement: Supplementary Figure S6 [file cddis2017270x6.tif]
