# Supplementary material for: Cdc20 directs proteasome-mediated degradation of the tumor suppressor SMAR1 in higher grades of cancer through the anaphase promoting complex
Source: Cell Death Dis. 2017 Jun 15;8(6):e2882–. doi: 10.1038/cddis.2017.270 (PMC5520925; doi:10.1038/cddis.2017.270)
Supplement: Supplementary Figure Legends [file cddis2017270x7.docx]

Figure S1: Endogenous level of SMAR1 is stabilized upon MG132 treatment. (a) Endogenous level of SMAR1 is stabilized upon MG132 treatment. Higher grades of breast cancer cell lines (MCF7: Luminal A, MDA-MB-231: Triple negative breast cancer cell line, basal B, NCI-ADR-RES: Luminal A and T47D: Luminal A) ^22- 24^ were treated with or without 10 μM MG132 for 6 h. Cells lysates were analysed by western blotting using indicated antibodies. (b) Quantification of SMAR1 level through densitometry analysis of immunoblots in panel (a). SMAR1 protein levels were measured using densitometry analysis and normalized against tubulin level. Normalized level of SMAR1 of untreated sample was taken as 1. (c) Relative mRNA level of SMAR1 in the absence and presence of MG132. SMAR1 mRNA levels were measured using RT-qPCR assay and normalized against GAPDH mRNA. (d) SMAR1 is regulated at proteasomal level in HCT116 and Hela cells.

Figure S2: Graphical representation of SMAR1 half-life in MCF7 and MDA-MB-231 cell lines. Expression of SMAR1 in figure 2f was quantified through densitometry analysis, normalized with expression of tubulin and normalized expression at zero h was taken as 100%.

Figure S3: The identified D-boxes were conserved through most of the species.

Figure S4: (a) SMAR1 is stabilized in T47D (Luminal A) and MDA-MB-231 (Triple negative) cells following Cdc20 depletion. (b) SMAR1 mRNA levels were measured using RT-qPCR assay and normalized against GAPDH mRNA. (c) Knockdown of APC2 led to lower the level of polyubiquitylated SMAR1. Cells stably expressing NS control and APC2 shRNA were treated with 10 μM MG132 for 6 h, lysates were immunoprecipitated for SMAR1 and immunoprecipitates were blotted for K48 linked ubiquitin.

Figure S5: Interaction of SMAR1 and Cdc20 increased upon JNK inhibition. SMAR1 was immunoprepitated from lysates of cells treated with or without 30 nM JNK inhibitor for 12 h. Cdc20 was cheeked in the immunoprecipitates.

Figure S6:Cdc20 promotes cell motility through degradation of SMAR1. (**a**) Immunoblot analysis for indicated proteins in wild type and different knockdown cells. (**b**) Cells were transfected with indicated plasmids for 36 h and transfected cells then were scratched by 200 μl tip and kept at 37°C, CO_2_ incubator and imaged at different time intervals. Histogram shows percentage of cells migrated as a mean ± SE. An *asterisk* indicates the significant difference (*, *p*<0.05) calculated by one way ANOVA test and ns represents not significant. (**c**) Cells transfected with plasmids of indicated genes for 24 h. Transfected cells were then starved for overnight and the seeded on the upper chamber of transwell chamber. After 12 h of seeding, invaded cells were stained with crystal violet and counted. Histogram shows the percentage of cells invaded as a mean ± SE. An *asterisk* indicates the significant difference (*, *p*<0.05) calculated by one way ANOVA test (**d**) Immunoblot analysis showed the expression level of SMAR1 in the transfected cells as in panel (b) and (c).
